# Clinical Characteristics and Prognosis of Adult Orbital Cellulitis in a Tertiary General Hospital

**DOI:** 10.1155/2020/8889341

**Published:** 2020-12-08

**Authors:** Bangtao Yao, Yuhua Ding, Xiaogui Zhao, Bei Wang, Gang Liu, Fei Wang

**Affiliations:** ^1^Department of Ophthalmology, Lishui District People's Hospital, Nanjing, Jiangsu Province, China; ^2^Department of Ophthalmology, Lishui Branch of Southeast University Affiliated Zhongda Hospital, Nanjing, Jiangsu Province, China; ^3^Department of Ophthalmology, Jiangsu Province Hospital, Nanjing, Jiangsu Province, China; ^4^Department of Ophthalmology, The First Affiliated Hospital of Nanjing Medical University, Nanjing, Jiangsu Province, China

## Abstract

**Purpose:**

Adult orbital cellulitis (OC) occurs relatively rarely, and comprehensive studies that retrospectively evaluate OC are lacking. Here, we aimed to examine the clinical characteristics and prognosis of OC in a tertiary general hospital.

**Methods:**

Between October 2010 and May 2019, patients presenting with clinical symptoms of OC in a tertiary general hospital were analyzed in this retrospective study. Twenty-six cases were identified for a detailed review. In these cases, 16 males and 10 females were diagnosed with orbital cellulitis by clinical characteristics and multimodal examinations. We divided patients into three groups: (1) patients secondary to rhinosinusitis, (2) patients secondary to endogenous infection(s) without endophthalmitis, and (3) patients secondary to endophthalmitis. For each group, age, gender, eye type, combined systemic diseases, clinical presentation, leukocyte count, blood culture, diagnostic imaging, therapeutic methods, length of stay, time of postoperation, and patient prognosis were analyzed in detail.

**Results:**

There were no significant differences regarding age, gender, preoperative leukocyte count, exophthalmia, blood culture, treatment, or visual changes within the three groups (*P* < 0.05). There were significant differences, however, in postoperative leukocyte count and ophthalmoplegia between the three groups (*P* < 0.05). The preoperative and postoperative logarithms of the minimum resolution angle scored by the best-corrected visual acuity (LogMAR BCVA) of group 3 were statistically significant compared to group 1 and group 2 (*P* < 0.05).

**Conclusion:**

We confirmed that the prognosis of OC patients combined with systemic diseases was poor. Patients with OC secondary to endophthalmitis infrequently experience ophthalmoplegia; however, these patients still have poor visual outcomes. Patients cultured positive for *Klebsiella pneumoniae* infection may not be associated with liver abscess. The level of leukocytes may indicate the condition of the disease.

## 1. Introduction

Orbital cellulitis (OC) is an urgent and deadly inflammation of the orbital soft tissue. OC is more common in children and relatively rare in adults [1]. Patients often present with blepharedema, ptosis, exophthalmia, ophthalmoplegia, ocular pain, and visual impairment [2]. OC can spread to the adjacent tissue or the cavernous sinus, leading to cavernous sinus thrombophlebitis, and endanger the patient's life [3]. Computed tomography (CT) scans or magnetic resonance imaging (MRI) are the standard tools for diagnosing OC, defining the extent of the infection [4] and identifying the size and location of the orbital abscess [2].

Several reports suggested that sinus inflammation, endophthalmitis, or endogenous infection metastasis could cause OC [5–7]. To the best of our knowledge, however, the available retrospective and comprehensive studies regarding the clinical characteristics and prognosis of adult OC in the literature are lacking. This article aims to describe the detailed clinical features and prognosis of adult OC in a tertiary general hospital over a 10-year period.

## 2. Materials and Methods

### 2.1. Study Design

This study was designed as a retrospective review of the medical records of 26 adult patients diagnosed with OC. The patients were admitted to a tertiary general hospital between October 2010 and May 2019. Children patients with OC were excluded from the review. Age, gender, eye type, combined systemic diseases, clinical presentation, leukocyte count, blood culture, CT or MRI, therapeutic methods, length of stay, time of postoperation, and prognosis are all described in detail.

This study was designed and conducted in accordance with the Declaration of Helsinki and was submitted to the appropriate review board. All participating patients provided informed consent for this study.

### 2.2. Statistical Analysis

We collected data from the patients' clinical records, and they were processed with SPSS (Statistical Package for Social Sciences, version 19.0, IBM). For categorical variables, Pearson's *χ*^2^ or Fisher's exact test was used; for continuous variables, the Kruskal–Wallis test was used. Two-tailed tests of significance were performed, and *P* values <0.05 were regarded as statistically significant.

## 3. Results

### 3.1. Demographics

The adult patients, 16 males and 10 females that were diagnosed with OC, had a median age of 48 years (range 32.75–66.50). All patients presented with OC in only one eye, with 14 cases that occurred in the right eye and 12 cases that occurred in the left eye. The patients were divided into three groups according to etiology: (1) patients secondary to rhinosinusitis, (2) patients secondary to endogenous infection(s) without endophthalmitis, and (3) patients secondary to endophthalmitis. In the six cases comprising group 1, the instances of OC were split evenly between the right and left eyes. Group 2 (*n* = 3) included two right eye cases and one left eye case. Finally, in group 3 (*n* = 17), nine and eight cases presented in the right and left eyes, respectively (Tables 1–3). There were no significant differences in age or gender among the three groups (*P* < 0.05).

### 3.2. Clinical Presentation

Group 1 and group 2 patients presented with blepharedema and moderately reduced visual acuity. Group 1 had five cases with exophthalmia and five cases with ophthalmoplegia, while group 2 had two cases with exophthalmia and two cases with ophthalmoplegia, with one patient presenting with both (Tables 1 and 2). The patients in group 3 presented with blepharedema and severe visual impairment: 11 cases with exophthalmia and 5 cases with ophthalmoplegia. The best-corrected visual acuity (BCVA) of 15 cases presented with no light perception (NLP), 1 case had LP, and 1 case had BCVA of 2/20 (Table 3).

### 3.3. Combined Systemic Diseases

OC does not always present alone. Two cases were complicated with diabetes in group 1, and 1 case was secondary to T-cell lymphoma. In group 2, two cases were complicated with iron-deficiency anemia or secondary to acute lymphocytic leukemia. Five cases were caused by trauma in group 3. Furthermore, 3 cases were complicated with liver or cervical abscesses, and 10 cases were complicated with other systemic diseases including sepsis, diabetes, hypertension, organ failure, or hematopathy (Tables 1–3).

### 3.4. Leukocyte Counts

Routine blood examinations were performed on all patients, and leukocyte counts were analyzed. The normal value of leukocyte counts was 4.0–10.0 × 1000 uL. The leukocyte counts of 6 patients, 3 patients, and 16 patients were abnormal before the treatment in groups 1, 2, and 3, respectively. While after treatment, the leukocyte counts of 5 patients and 16 patients returned to normal in groups 1 and 3, respectively. However, the total leukocyte counts of 3 patients in group 2 remained abnormal consistently (Tables 1–3).

### 3.5. Blood Cultures

Blood cultures were obtained from all patients; the bacterial culture and fungal culture were both ordered in the blood culture, and three of them were positive. Two cases tested positive for *Klebsiella pneumoniae,* and one case was positive for *Pseudomonas aeruginosa.* The fungal cultures were all negative (Tables 2 and 3).

### 3.6. CT or MRI

Each patient received CT initially. MRI was performed in ten patients (Tables 1–3). CT scans revealed swollen orbital soft tissue, increased fat density, and opacification of the involved sinuses in the patients with sinusitis. Two cases presented bony destruction, and thickening of the associated muscles was present in eight cases. Optic nerve involvement was demonstrated in one case. In addition, we observed one case combined with cavernous sinus thrombophlebitis.

MRI revealed ill-defined infiltration of orbital fat. Only four cases demonstrated an extremely hyperintense signal representing orbital abscess formation when analyzed by diffusion-weighted imaging (DWI) and the corresponding hypointense signal on the apparent diffusion coefficient (ADC).

### 3.7. Length of Stay

In group 1, the mean hospital stay was 17.17 ± 9.87 days, and the mean time to discharge after operation was 12.33 ± 9.45 days. For group 2, the mean hospital stay was 19.33 ± 3.06 days, and the mean time to discharge after operation was 2.00 ± 3.46 days. Finally, in group 3, the mean hospital stay was 11.94 ± 5.73 days with a mean time to discharge after operation of 5.71 ± 3.80 days (Tables 1–3).

### 3.8. Therapeutic Methods and Prognosis

All patients diagnosed with OC were treated with active treatments once admitted to the hospital, including systemic and topical antibiotics, and further therapeutic plans were made according to the patients' conditions.

Three cases in group 1 were treated with endoscopic sinus surgery, while another three cases were treated conservatively. Only one patient could not visualize hand motion at the time of hospitalization and had NLP at the time of discharge. In this case, we considered that inflammation spread to the optic nerve. One case in group 2 was treated with abscess incision, and the other two cases were treated with conservative treatment.

Six cases in group 3 were treated with intravitreal injection of vancomycin and cefazolin. Five and two cases were treated with the evisceration of eye contents and enucleation, respectively. Abscess incision was done in two cases, and three cases were treated with conservative treatment. Unfortunately, all patients had a BCVA of NLP (Tables 1–3).

Microbial cultures of the drained abscesses from three patients were performed, and *Klebsiella pneumoniae* was identified and cultured in one patient. In two patients, there was no growth reported.

### 3.9. Group Comparisons

There were no significant differences in preoperative leukocyte count, exophthalmia, blood culture, treatment, and visual changes among the three groups (*P* < 0.05). There were significant differences in postoperative leukocyte count and ophthalmoplegia between the three groups (*P* < 0.05). The preoperative and postoperative minimum resolution angles in logarithm best-corrected visual acuity (LogMAR BCVA) of group 3 were statistically significant compared with group 1 and group 2 (*P* < 0.05) (Tables 4 and 5).

## 4. Discussion

OC can be divided into anterior orbital septum cellulitis and posterior orbital septum cellulitis [8]. A variety of reasons cause OC. Firstly, inflammation of ethmoid, maxillary, frontal, or sphenoid sinuses can lead to OC [5]. Secondly, reports indicate that endophthalmitis commonly causes OC [6]. Thirdly, some OC patients had secondary endogenous infection metastasis [7]. In immunocompromised patients or in patients with systemic diseases, such as leukemia, T-cell lymphoma, sepsis, and diabetes, OC more easily leads to endogenous and secondary infections [9].

OC resulting from rhinosinusitis occurs most frequently in children and rarely in adults [2]. Contradicting this finding, however, Siedek et al. found that 75% of rhinosinusitis-caused OC patients in their study were adults [5]. The anatomy of the sinus lies close to the orbit. As a result, sinus inflammation may spread directly to the orbit through the bone or indirectly through the valveless venous plexus surrounding the orbit and the sinuses [10]. Spread through the ethmoid sinusitis was the most common because of the thin ethmoid bone [2]. According to Stammberger's classification, OC is the most serious ocular complication, secondary to rhinosinusitis (stage IV) [11]. Reports suggested that stages I and II can be treated conservatively, while stages III and IV were recommended to receive surgery [5]. In our group 1 of all adult patients, five patients belonged to stage IV, 60% (*n* = 3) of which were treated with endoscopic sinus surgery. Of those who received surgery, 66.7% (*n* = 2) recovered, while one patient with T-cell lymphoma deteriorated.

OC also can be caused by endophthalmitis or panophthalmitis. While the underlying mechanism remains unclear, it is generally assumed that intraocular inflammation can spread to the periocular tissues, resulting in orbital cellulitis [6, 12]. In traumatic endophthalmitis, the wound may implant the pathogenic bacteria directly in the periocular or orbital tissues. By a similar mechanism, cataract, strabismus, or retinal detachment surgery can also lead to OC [6, 13, 14]. Endogenous endophthalmitis is especially prone to occur in immunocompromised patients or patients with severe systemic diseases. In these cases, the primary origins of the infection cannot be found [9].

Endophthalmitis patients with mild symptoms can be treated conservatively, but severe cases can be treated with intravitreal injection. In these instances, vancomycin and cefazolin have proven effective in controlling the inflammation [15]. If the disease still progresses, enucleation or evisceration surgery should be performed [16]. In our study, six cases in group 3 were treated with intravitreal injection (vancomycin and cefazolin), five cases were treated with evisceration of the eye contents, two cases were treated with enucleation, and three cases were treated conservatively. In total, 17 cases (100%) of OC with secondary endophthalmitis were effectively controlled. We considered that OC secondary to endophthalmitis could be controlled if active treatments are given.

*K. pneumoniae* is a small, packaged, Gram-negative bacterium [17], and secondary endophthalmitis complicated with a liver abscess is considered a clinical syndrome closely associated with diabetes in Asians [18]. In our study, two patients cultured positive for *K. pneumoniae* infection by blood culture. They were found with cervical abscess and sepsis, however, which were not associated with liver abscess. On the contrary, the blood cultures of the two liver abscess patients were negative for pathogenic bacteria.

OC in only three patients was caused by endogenous infection without endophthalmitis. In these instances, one case was complicated with acute lymphocytic leukemia, and one situation was complicated with iron-deficiency anemia and ulcerative colitis. In the first patient, the blood culture was positive for *P. aeruginosa,* and the patient had complications with sepsis during the hospitalization period. The condition deteriorated eventually even after timely treatment.

Routine blood tests showed evidence of an increase in leukocyte count in most OC patients [2]. In our study, comparisons among the three groups illustrated that postoperative leukocyte counts of patients in group 2 changed more dramatically than the other two groups. We also noticed that the incidence of ophthalmoplegia in group 3 was less than that of other groups, indicating that OC with secondary endophthalmitis rarely invaded extraocular muscles (29.4%), which have not been reported in the literature. Furthermore, in comparing the preoperative and postoperative LogMAR BCVA among the three groups, we concluded that patients in group 3 had poorer visual outcomes than any other group because all patients ended up with the BCVA of NLP. Endophthalmitis is a severe disease that can result in serious visual impairment and even blindness. Therefore, we concluded that the poorer vision in group 3 is attributable to endophthalmitis.

In our study, bacterial and fungal blood cultures were performed. Three patients were positive for bacteria (11.5%), while the other patients were negative. Fungal infections are common in adult OC patients with diabetes [10]. However, in the present study, the results of fungal cultures were all negative, even in the patients combined with systemic diseases, such as diabetes, leukemia, T-cell lymphoma, and sepsis. Similarly, in a study by Hsu J et al., blood cultures were the most frequently collected but were the least likely to be positive (17.6%) [19]. We speculated that the results were negative due to the antibiotic treatment prior to blood collection. In addition, leukocyte counts are still a suitable parameter for diagnosing infection. However, the relationship between the prognosis of infection and leukocyte has been rarely described [20]. In our study, the routine blood tests showed evidence of elevated leukocyte count in all the patients except for 2, one complicated with acute lymphocytic leukemia and the other with T-cell lymphoma. The leukocyte counts returned to normal when the patients recovered. However, the counts of the two patients with acute lymphocytic leukemia and T-cell lymphoma remained abnormal. This finding is consistent with the deterioration of their disease, so we concluded that the leukocyte counts might indicate the recovery or deterioration of the disease.

There were several limitations to this study. Firstly, this retrospective study had a small sample size. Secondly, the patient had been treated with antibiotics at the time of blood culture collection. As a result, the positive rate is relatively low. Thirdly, the patients included were all adults, so this study is unable to describe the differences in clinical characteristics and prognosis between adults and children with OC.

## 5. Conclusions

We confirmed that OC could be well controlled if timely treatments are given. The prognosis of orbital cellulitis patients combined with severe systemic diseases, however, was generally poor. While patients with orbital cellulitis secondary to endophthalmitis infrequently experience ophthalmoplegia, these patients still have poor visual outcomes. Patients cultured positive for *Klebsiella pneumoniae* infection may not be associated with liver abscess. Furthermore, the leukocyte levels may indicate the condition of the disease. More data by multiple centers are required to understand the clinical characteristics and prognosis of OC better.

## Figures and Tables

**Table 1:** Clinical data of 6 patients secondary to rhinosinusitis.

No.	Age/gender/laterality	Sinuses involved	Clinical presentation	Systemic diseases	Leukocyte count (initial vs. final)	Blood culture	CT or MRI	Treatment	Length of stay (days)	Time of postoperation (days)	Initial BCVA	Final BCVA	Prognosis
1	39/F/OD	Ethmoid, sphenoid maxillary	Blepharedema, ophthalmoplegia, exophthalmia	T-cell lymphoma	1.5–2.6	Negative	CT MRI	Endoscopic sinus surgery	32	23	20/40	20/100	Deteriorated
2	73/M/OS	Ethmoid, maxillary	Blepharedema, ophthalmoplegia, exophthalmia	Diabetes hypertension	13.5–7.86	Negative	CT	Endoscopic sinus surgery	23	5	HM	NLP	Recovered
3	27/F/OS	Ethmoid, maxillary frontal	Blepharedema, ophthalmoplegia, exophthalmia	N/A	12.7–7.54	Negative	CT MRI	Endoscopic sinus surgery	10	9	20/50	20/50	Recovered
4	34/F/OD	Ethmoid, maxillary	Blepharedema	N/A	11.5–5.6	Negative	CT	Conservative	4	N/A	20/60	20/60	Recovered
5	63/F/OD	Ethmoid, maxillary frontal	Blepharedema, ophthalmoplegia, exophthalmia	Diabetes	10.81–8.5	Negative	CT	Conservative	15	N/A	20/20	20/20	Recovered
6	46/M/OS	Sphenoid	Blepharedema, ophthalmoplegia, exophthalmia	Cavernous sinus thrombophlebitis	3.2–6.9	Negative	CT MRI	Conservative	19	N/A	20/100	20/60	Improved

NLP: no light perception; BCVA: best-corrected visual acuity; N/A: not applicable; CT: computed tomography; MRI: magnetic resonance imaging; HM: hand motion.

**Table 2:** Clinical data of 3 patients secondary to endogenous infection(s) without endophthalmitis.

No.	Age/gender/laterality	Clinical presentation	Systemic diseases	Leukocyte count (initial vs. final)	Blood culture	CT or MRI	Treatment	Length of stay (days)	Time of postoperation (days)	Initial BCVA	Final BCVA	Prognosis
1	26/M/OD	Blepharedema, ophthalmoplegia, exophthalmia	Iron-deficiency anemia	22.85–10.54	Negative	CT MRI	Abscess incision	22	6	20/100	20/60	Recovered
2	50/F/OS	Blepharedema	Rheumatism	11.16–10.57	Negative	CT	Conservative	16	N/A	20/20	20/20	Recovered
3	28/M/OD	Blepharedema, ophthalmoplegia, exophthalmia	Acute lymphocytic leukemia, sepsis	1.64–0.24	Pseudomonas aeruginosa	CT MRI	Conservative	20	N/A	20/40	20/60	Deteriorated

BCVA: best-corrected visual acuity; N/A: not applicable; CT: computed tomography; MRI: magnetic resonance imaging.

**Table 3:** Clinical data of 17 patients secondary to endophthalmitis.

No.	Age/gender/laterality	Etiology	Clinical presentation	Systemic diseases	Leukocyte count (initial vs. final)	Blood culture	CT or MRI	Treatment	Length of stay (days)	Time of postoperation (days)	Initial VA	Final VA	Prognosis
1	44/M/OD	Traumatic	Blepharedema, ophthalmoplegia, exophthalmia	N/A	10.85–6.64	Negative	CT	Intravitreal injection (twice)	9	4	NLP	NLP	Recovered
2	41/M/OD	Endogenous	Blepharedema, exophthalmia	Diabetes	13.8–8.6	Negative	CT	Intravitreal injection (twice)	28	12	LP	NLP	Recovered
3	87/M/OD	After cataract surgery	Blepharedema, ophthalmoplegia, exophthalmia	Pulmonary embolism	12.7–5.93	Negative	CT	Evisceration of eye contents	7	7	NLP	NLP	Recovered
4	28/F/OS	Endogenous	Blepharedema, exophthalmia	Diabetes hypertension uremia	12.1–9.98	Negative	CT	Evisceration of eye contents	12	6	NLP	NLP	Recovered
5	64/F/OS	Traumatic	Blepharedema, exophthalmia	Hypertension	10.09–4.70	Negative	CT MRI	Evisceration of eye contents	12	7	NLP	NLP	Recovered
6	28/M/OD	Traumatic	Blepharedema, exophthalmia	N/A	14.85–5.6	Negative	CT	Intravitreal injection (once)	10	10	NLP	NLP	Recovered
7	65/M/OS	Corneal perforation	Blepharedema	N/A	10.18–6.2	Negative	CT	Enucleation	12	4	NLP	NLP	Recovered
8	29/M/OD	Traumatic	Blepharedema	N/A	23.08–13.7	Negative	CT MRI	Intravitreal injection (once)	16	12	20/200	NLP	Recovered
9	60/M/OD	Endogenous	Blepharedema, exophthalmia	Diabetes hypertension cervical abscess	12.5–8.7	*Klebsiella pneumonia*e	CT MRI	Abscess incision, vitrectomy, intravitreal injection	19	4	NLP	NLP	Recovered
10	87/M/OS	Endogenous	Blepharedema, ophthalmoplegia, exophthalmia	Pulmonary encephalopathy	15.4–8.25	Negative	CT	Evisceration of eye contents	6	4	NLP	NLP	Recovered
11	63/F/OS	Endogenous	Blepharedema, exophthalmia	N/A	9.1–7.4	Negative	CT	Vitrectomy intravitreal injection	9	7	NLP	NLP	Recovered
12	65/M/OD	Endogenous	Blepharedema, ophthalmoplegia, exophthalmia	COPD hypertension	17.4–8.46	Negative	CT MRI	Abscess incision	5	5	NLP	NLP	Recovered
13	71/F/OS	Endogenous	Blepharedema, ophthalmoplegia, exophthalmia	Liver abscess	21.84–7.8	Negative	CT	Enucleation	17	10	NLP	NLP	Recovered
14	34/M/OD	Traumatic	Blepharedema	N/A	12.46–8.21	Negative	CT	Evisceration of eye contents	10	5	NLP	NLP	Recovered
15	77/M/OS	Endogenous	Blepharedema	Liver abscess hypertension cerebral infarction	12.92–9.0	Negative	CT	Conservative	9	N/A	NLP	NLP	Recovered
16	45/M/OS	Endogenous	Blepharedema	Sepsis hypertension	13.05–8.91	*Klebsiella pneumonia*e	CT	Conservative	15	N/A	NLP	NLP	Recovered
17	73/F/OD	Corneal perforation	Blepharedema	N/A	12.6–6.94	Negative	CT MRI	Conservative	7	N/A	NLP	NLP	Recovered

NLP: no light perception; BCVA: best-corrected visual acuity; N/A: not applicable; COPD: chronic obstructive pulmonary disease; CT: computed tomography; MRI: magnetic resonance imaging.

**Table 4:** Demographics and clinical presentation of 26 patients.

Group No.	Age (years) (*n*)			Gender (*n*)		Ophthalmoplegia (*n*)		Exophthalmia (*n*)		Leukocyte counts (*n*)				Blood culture (*n*)		Treatment (*n*)		BCVA (initial vs. final) (*n*)		
	18–45	46–59	>60	Male	Female	Yes	No	Yes	No	Initial		Final		Positive	Negative	Conservation	Operation	Improved	Unchanged	Decreased
										Normal	Abnormal	Normal	Abnormal							
1	6	3	1	2	2	4	5	1	5	1	0	6	5	1	0	6	3	3	1	4	1
2	3	2	1	0	2	1	2	1	2	1	0	3	0	3	1	2	2	1	1	1	1
3	17	7	0	10	12	5	5	12	11	6	1	16	16	1	2	15	3	14	2	0	15
*P* value	—	0.136			0.231		0.047		0.822		1.000		0.003		0.425		0.138		0.086		

BCVA: best-corrected visual acuity.

**Table 5:** The BCVA and length of stay of 26 patients.

Group	Cases	No.	Initial BCVA	Final BCVA	Length of stay (days)	Time of postoperation (days)
1	6	1	20/40	20/100	32	23
		2	HM	NLP	23	5
		3	20/50	20/50	10	9
		4	20/60	20/60	4	N/A
		5	20/20	20/20	15	N/A
		6	20/100	20/60	19	N/A

2	3	7	20/100	20/60	22	6
		8	20/20	20/20	16	N/A
		9	20/40	20/60	20	N/A

3	17	10	NLP	NLP	9	4
		11	LP	NLP	28	12
		12	NLP	NLP	7	7
		13	NLP	NLP	12	6
		14	NLP	NLP	12	7
		15	NLP	NLP	10	10
		16	NLP	NLP	12	4
		17	20/200	NLP	16	12
		18	NLP	NLP	19	4
		19	NLP	NLP	6	4
		20	NLP	NLP	9	7
		21	NLP	NLP	5	5
		22	NLP	NLP	17	10
		23	NLP	NLP	10	5
		24	NLP	NLP	9	N/A
		25	NLP	NLP	15	N/A
		26	NLP	NLP	7	N/A

*P* value			1 vs. 2 : 1.000	1 vs. 2 : 1.000	0.083	0.380
			1 vs. 3 : 0.000	1 vs. 3 : 0.000		
			2 vs. 3 : 0.006	2 vs. 3 : 0.005		

NLP: no light perception; BCVA: best-corrected visual acuity; N/A: not applicable.

## Data Availability

The data used to support the findings of this study are included within the article.

## Abbreviations

BCVA: Best-corrected visual acuity
CT: Computed tomography
LogMAR: Minimum resolution angle in logarithm
MRI: Magnetic resonance imaging
NLP: No light perception
OC: Orbital cellulitis.

## References

[1] Sciarretta V., Demattè M., Farneti P. (2017). Management of orbital cellulitis and subperiosteal orbital abscess in pediatric patients: a ten-year review. *International Journal of Pediatric Otorhinolaryngology*.

[2] Tsirouki T., Dastiridou A. I., Ibánez flores N. (2018). Orbital cellulitis. *Survey of Ophthalmology*.

[3] Gupta R., Patadia D., Velayudhan V., Buchnea D. (2017). Orbital cellulitis, cavernous sinus thrombosis, internal jugular vein thrombus, and clival osteomyelitis secondary to acute sinusitis. *American Journal of Respiratory and Critical Care Medicine*.

[4] Jabarin B., Eviatar E., Israel O., Marom T., Gavriel H. (2018). Indicators for imaging in periorbital cellulitis secondary to rhinosinusitis. *European Archives of Oto-Rhino-Laryngology*.

[5] Siedek V., Kremer A., Betz C. S., Tschiesner U., Berghaus A., Leunig A. (2010). Management of orbital complications due to rhinosinusitis. *European Archives of Oto-Rhino-Laryngology*.

[6] Decock C., Claerhout I., Kestelyn P., Van Aken E. H. (2010). Orbital cellulitis as complication of endophthalmitis after cataract surgery. *Journal of Cataract & Refractive Surgery*.

[7] Hornblass A., To K., Coden D. J., Ahn-Lee S. (1989). Endogenous orbital cellulitis and endogenous endophthalmitis in subacute bacterial endocarditis. *American Journal of Ophthalmology*.

[8] Ivanišević M., Ivanišević P., Lešin M. (2019). Epidemiological characteristics of orbital cellulitis among adult population in the Split region, Croatia. *Wien Klin Wochenschr*.

[9] Collazos J., de la Fuente B., García A. (2018). Cellulitis in adult patients: a large, multicenter, observational, prospective study of 606 episodes and analysis of the factors related to the response to treatment. *PLoS One*.

[10] Gupta S., Goyal R., Gupta R. K. (2013). Clinical Presentation and outcome of the orbital complications due to acute infective rhino sinusitis. *Indian Journal of Otolaryngology and Head & Neck Surgery*.

[11] Stammberger H. (1993). Komplikationen entzündlicher Nasennebenhöhlenerkrankungen einschließlich iatrogen bedingter Komplikationen. *Referate*.

[12] Guedira G., Taright N., Blin H. (2018). Clostridium perfringens panophthalmitis and orbital cellulitis: a case report. *BMC Ophthalmology*.

[13] Kim E. C., Kim M. S., Kang N. Y. (2013). Fungal corneal ulcer and bacterial orbital cellulitis occur as complications of bacterial endophthalmitis after cataract surgery in an immunocompetent patient. *Seminars in Ophthalmology*.

[14] Kim J. M., Megalla M., Howard M., Sinard J., Pointdujour-Lim R. (2018). Orbital cellulitis with choroidal detachment following strabismus surgery in an adult. *Journal of American Association for Pediatric Ophthalmology and Strabismus*.

[15] Nentwich M. M., Yactayo-Miranda Y., Schwarzbach F., Wolf A., Kampik A., Mino de Kaspar H. (2014). Endophthalmitis after intravitreal injection. *Retina*.

[16] Lee K. M., Han S. C., Ho S. Y. M., Kim J. T., Kim Y. H. (2013). Blindness resulting from orbital cellulitis following rhinoplasty. *Journal of Plastic, Reconstructive & Aesthetic Surgery*.

[17] Yen C. H., Wu S. Y., Liao Y. L. (2018). Klebsiella pneumoniae orbital cellulitis: clinical manifestations and outcomes in a tertiary medical center in Taiwan. *Journal of Ophthalmology*.

[18] Davies B. W., Fante R. G. (2016). Concurrent endophthalmitis and orbital cellulitis from metastatic klebsiella pneumonia liver abscess. *Ophthalmic Plastic and Reconstructive Surgery*.

[19] Hsu J., Treister A. D., Ralay Ranaivo H., Rowley A. H., Rahmani B. (2019). Microbiology of pediatric orbital cellulitis and trends in methicillin-resistant staphylococcus aureus cases. *Clinical Pediatrics*.

[20] Lavoignet C.-E., Le Borgne P., Chabrier S. (2019). White blood cell count and eosinopenia as valuable tools for the diagnosis of bacterial infections in the ED. *European Journal of Clinical Microbiology & Infectious Diseases*.

